# A Review on Preparation of Betulinic Acid and Its Biological Activities

**DOI:** 10.3390/molecules26185583

**Published:** 2021-09-14

**Authors:** Hanghang Lou, Hao Li, Shengliang Zhang, Hongyun Lu, Qihe Chen

**Affiliations:** Department of Food Science and Nutrition, Zhejiang University, Hangzhou 310058, China; louhanghang@zju.edu.cn (H.L.); lhh20210901@126.com (H.L.); 12113064@zju.edu.cn (S.Z.); luhongyun@zju.edu.cn (H.L.)

**Keywords:** betulinic acid, preparation, biological activities, triterpenes

## Abstract

Betulinic acid, a pentacyclic triterpene, is distributed in a variety of plants, such as birch, eucalyptus and plane trees. It shows a wide spectrum of biological and pharmacological properties, such as anti-inflammatory, antibacterial, antiviral, antidiabetic, antimalarial, anti-HIV and antitumor effects. Among them, the antitumor activity of betulinic acid has been extensively studied. However, obtaining betulinic acid from natural resources can no longer meet the needs of medicine and nutrition, so methods such as chemical synthesis and microbial biotransformation have also been used to prepare betulinic acid. At the same time, with the development of synthetic biology and genetic engineering, and the elucidation of the biosynthetic pathways of terpenoid, the biosynthesis of betulinic acid has also been extensively researched. This article reviews the preparation of betulinic acid and its pharmacological activities, in order to provide a reference for the research and utilization of betulinic acid.

## 1. Introduction

**Figure 1:**
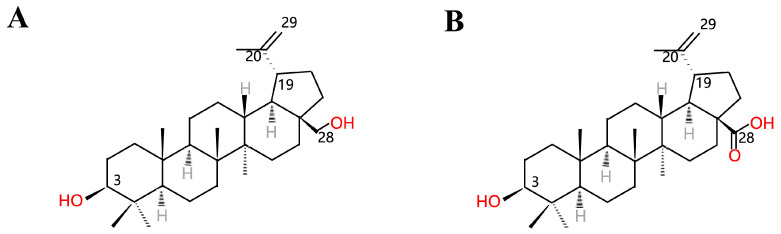
Structures of (**A**) betulin and (**B**) betulinic acid.

Triterpenes are a chemically diverse class of compounds, and many among them are of interest from a human health perspective. Betulinic acid (BA, 3-beta-hydroxy-lup20(29)-en-28-oic acid, Figure 1B) is a pentacyclic lupane-type triterpenoid that is widely distributed throughout the plant kingdom [1,2]. The chemical structures of lupane-type triterpenoids are shown in Figure 1. Among these compounds, recently, BA has gained considerable interest owing to a variety of biological and pharmacological activities that have been ascribed to this compound, including anti-inflammatory, antibacterial, antiviral, antidiabetic, antimalarial, anti-HIV and antitumor effects [1]. Because of its specific cytotoxicity against tumor cells, BA is considered a future promising antitumor compound [3,4,5]. Interestingly, despite its great potential for clinical applications, the insufficient supply of BA in its natural hosts is a major challenge in commercializing this compound. Though birch bark is the major plant source for extracting BA, the minute amount of BA in its tissues has limited its production on a large scale for the market [6]. Thus, developing more methods to prepare this compound is a major research area. Chemical synthesis based on betulin (Figure 1A) as the precursor has been reported generally [7,8,9]. Recently, microbial biotransformation became another approach for converting betulin to BA, but the conversion efficiency is pretty low, and this approach is also limited by the supply of betulin [10]. Though the pharmaceutical and physiological importance of these triterpenoids is known, understanding of their biosynthetic pathways remains limited [11]. Rapid developments in metabolic engineering and synthetic biology provide alternative approaches for the increased production of natural products in microbial hosts [12,13,14,15]. The identification of newly identified microbes that convert betulin to BA is recently more intentioned [16], and metabolic engineering of key biosynthetic genes derived from plant sources has been reported as well. As a consequence, this review discusses recent developments in biotransformation and metabolic engineering for BA production. The purpose of this review is to describe recently published literature on the derived sources and production of BA and introduce its pharmacological activity. Furthermore, this review presents insights and strategies for the sustainable metabolic engineering of BA in multifarious transgenic organisms.

## 2. Preparation of Betulinic Acid

BA is generally derived from plant sources, such as birch, eucalyptus and plane trees. However, it is well known that the low concentration in plant sources has limited its research and application. To solve these problems, nowadays, more research is focused on the construction of new synthesis methods such as chemical synthesis, biotransformation, metabolic engineering, etc. Herein, we summarize the progress of BA preparation in recent times.

### 2.1. Extract Betulinic Acid from Plants

So far, BA has been mainly found in the outer bark of the white birch tree. The BA concentration in birch bark was found to be about 0.002–2%, and the yield depended on the extraction solvents and methods. Mukherjee et al. [17] used 70% ethanol to extract BA and obtained a yield of 23.76 mg/10 g birch bark. Kim et al. [18] obtained a comparatively low yield of BA (0.0021%) from birch bark by ultrasonic extraction. Ethyl acetate was also used in the maceration extraction of BA, and the yield reached 3.07 mg/g birch bark [19]. To overcome the environmental problems and the production of hazardous waste of the above methods, new environmentally and human-friendly technologies were implemented into BA extraction. As a result, the yield could reach 28.3 mg/10 g birch bark after optimization [20]. Additionally, BA was identified in several other botanical tissues. In 1948, BA was identified in the bark of the plane tree [21]. In 1999, Galgon et al. [22] conducted quantification of BA in the cork of the plane tree. The results showed that the concentration was about 3.3%. In a comparative study, supercritical fluid extraction using ethanol as the cosolvent of CO_2_ achieved a high BA extraction yield (4.34%) from the dried bark of the plane tree (*Platanus acerifolia* L.) and almost one-third of the organic consumption compared with solid–liquid extraction (SLE), ultrasound-assisted extraction (UAE) and pressurized liquid extraction (PLE) [23]. BA was also found to exist in the leaves, stem and bark of *Syzygium aromaticum* (L.) Merr. & L.M. Perry (SA), a tropical evergreen tree [24]. The botanical materials were first extracted in methanol by a Soxhlet extractor, and the extracts were dried into solid. The BA content of the leaf extracts of SA was higher than the content of the stem and bark extracts, which was 16.9 ± 0.9, 3.5 ± 0.2 and 3.8 ± 0.4 [24]. BA was also detected in *Eucalyptus* spp., an important fiber source for pulp and paper production. BA could be extracted from the biomass residues of the pulping industry [25,26,27,28] and also isolated from Lamiaceae plants [29,30]. Vietnamese researchers isolated BA from the aerial part of *Orthosiphon stamineus* using ethanol [29]. Machado et al. [30] isolated BA from the dried aerial parts of *Rosmarinus officinalis* L. Nyasse et al. [31] isolated BA from five *Uapaca* from Cameroon, and the concentration ranged from 1.5% to 1.9%. Chinese folk medicine, such as *Ziziphus jujube* Mill var. *spinosa* (Bunge) Hu ex. H.F. Chou, was also found as a source of BA [32,33]. Sun et al. [32] and Zhang et al. [33] isolated BA from sour jujube fruits and semen Ziziphi spinosae, respectively. Furthermore, Nigerian researchers isolated BA from the roots of *Tetracera potatoria* [34]. BA was obtained from *Souroubea sympetala* [35]. *Callistemon lanceolatus*, an Australian native plant and widely distributed in subtropical regions, was discovered harboring BA too [36]. Lin et al. [37] extracted BA from the aerial roots of *Avicennia marina*, which was a cosmopolitan species resident in tropical and subtropical regions.

### 2.2. Chemical Synthesis

**Figure 2:**
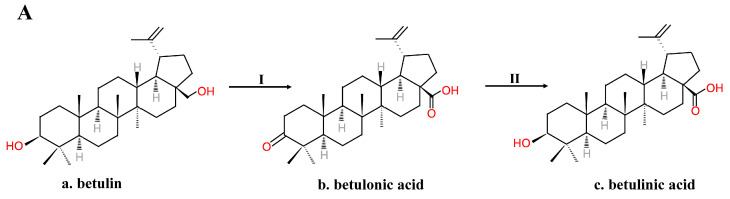

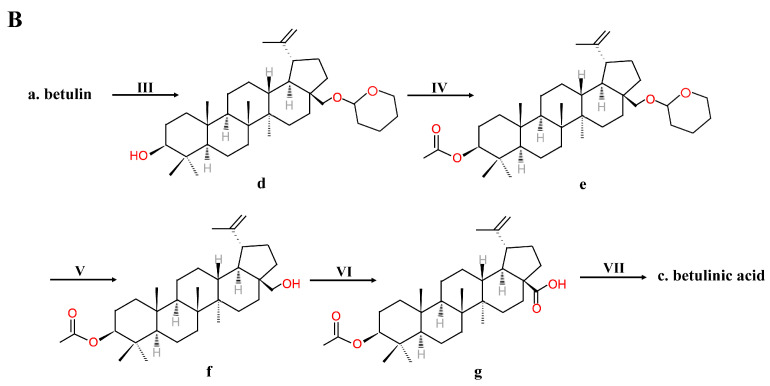
(**A**) Kim’s two-step method for obtaining BA from betulin. (**B**) Kim’s five-step method for obtaining b-isomer BA from betulin. I. Jones oxidation. II. Reduction. III. Primary alcohol THP esterification. IV. Acetylation of the secondary alcohol. V. Removing the THP ester. VI. Jones oxidation. VII. Removing the acetyl group.

Because of its various bioactivities, BA is in great need. However, extracting BA from botanical materials is time-consuming, not environmentally friendly and produces a low yield. Thus, extraction is not suitable for large-scale manufacturing. Chemical synthesis is another method to obtain BA. In 1997, Kim et al. [38,39] obtained BA by first oxidizing betulin using Jones reagent—for example, chromium trioxide (CrO_3_), sulfuric acid (H_2_SO_4_) and acetone at 0 ℃—and then reducing it using sodium borohydride in THF. The oxidation step oxidized the betulin into betulonic acid by oxidizing the primary carboxylic acid functionality and the secondary hydroxyl functionality to a keto functionality [38,39]. The reduction step only reduced the keto functionality of betulonic acid to hydroxyl functionality [38,39] (Figure 2A). To obtain only the b-isomer BA, a five-step synthesis without the reduction step was established [38,39] (Figure 2B). The primary alcohol of BA dissolved in CH_2_Cl_2_ was protected by esterification into a THP ether using dihydropyran (DHP) and pyridinium p-toluene sulfonate (PPTS), and then the secondary alcohol was acetylated using Ac_2_O and pyridine. The THP ether was selectively hydrolyzed and removed using methanol and PPTS, and Jones oxidation could turn the primary alcohol into carboxylic acid. To obtain b-isomer BA, the acetyl group was removed using K_2_CO_3_, methanol and H_2_O [38,39]. Gaudet et al. [40] added white birch bark directly with CrO_3_ absorbed in Al_2_O_3_ in CH_2_Cl_2_ and then used potassium permanganate for further oxidation to harvest BA. Russian scientists also created a method similar to Kim’s [9]. Betulin was oxidized to betulonic acid with a pyridine dichromate complex and acetic anhydride in dimethylformamide in a ratio of 2.5:1–3:1, and betulonic acid was reduced to alpha (5%) and beta (95%) isomers with sodium boron hydride in C2-4 alcohol [9]. Then, recrystallization of BA from C2-4 alcohol to form a natural isomer of BA from the alpha-isomer took place [9].

**Figure 3:**
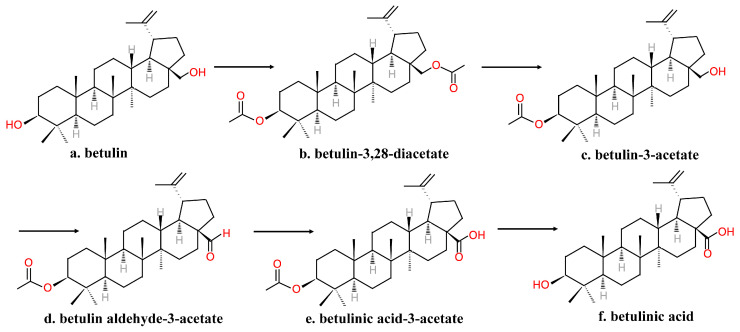
Krasutsky’s multistep method of manufacturing betulinic acid.

The reagents used in the above method were expensive and hazardous; in addition, the process is time-consuming. Thus, Krasutsky et al. [41,42] improved the method of manufacturing BA into one that requires less time and reagents that are less expensive and less toxic (Figure 3). Betulin was first acylated to betulin-3,28-diacetate by heating under reflux for 2–5 h in acetic anhydride and acetic acid. Then, betulin-3-acetate was obtained via selective deacetylation using aluminum isopropoxide. After, betulin-3-acetate was oxidized to betulin aldehyde-3-acetate with the use of palladium acetate, molecular sieves and oxygen in trifluoromethylbenzene and pyridine at 80–85 ℃ for 0.5–4 h. Further, betulin aldehyde-3-acetate was oxidized to betulinic acid-3-acetate with the use of oxygen and cobalt acetylacetonate in trifluoromethylbenzene at 60–65 ℃ for 0.5–2 h. Finally, the C-3 OH group was deprotected to provide BA [41,42].

**Figure 4:**
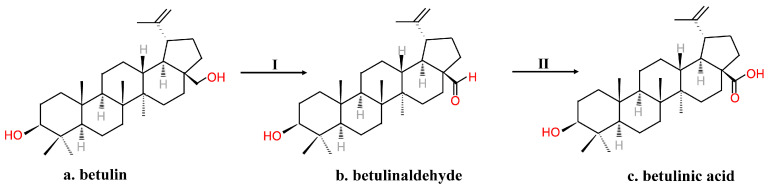
A short two-step procedure synthesizing betulinic acid from betulin. I. TEMPO, H+, electrochemical. II. [O].

However, the substrate was sensitive to degradation. The process needed several steps of protection and deprotection, and although the time needed for the process was reduced compared to Kim’s method, it was still time-consuming for commercial use. Menard et al. [8] subjected betulin to a short two-step synthesis to form BA (Figure 4). The C-28 OH group of substrates that dissolved in glacial acetic acid was first electrochemically oxidized to aldehyde using TEMPO. After isolation and purification, a mild oxidizing agent such as NaClO_2_ in t-butanol oxidized the aldehyde into BA [8].

### 2.3. Biotransformation Process

#### 2.3.1. Biotransformation by Fungi System

Obtaining BA from betulin catalyzed by cultured fungi is a possible method. Chen’s laboratory has conducted several studies on this [10,43,44,45]. Chen et al. [43] screened eight fungi for their capability to transform betulin into BA. Under the designed conditions, *Armillaria luteo-virens* Sacc QH (ALVS), *Aspergillus foetidus* ZU-G1 (AF) and *Aspergillus oryzae* (AO) showed a significantly increased effect on BA production [43]. In a further study comparing the effluence of different transformation conditions, ALVS was found to be the most effective strain for BA production, and the optimal condition was G2 (growing cell, preincubation for 3 days, then transformation for 6 days) with a high yield close to 28% [43]. Liu et al. [10] optimized the biotransformation conditions of *A*. *luteo-virens* Sacc ZJUQH100-6, a strain using low-energy N^+^ implantation mutated from *A*. *luteo-virens* Sacc QH, and obtained the predicted optimal of 9.32%, which was 174.53% of the nonoptimized condition. Fu et al. [44] used an ionic-liquid-containing system as the reaction medium of *A. luteo-virens* Sacc ZJUQH100-6 to produce BA and obtained a yield of 11.4%, which was higher than the PDA aqueous system, the production of which was 8.12%. *Cunninghamella blakesleeana* was also used in the biotransformation to obtain BA [45]. Feng et al. [45] discovered that *C*. *blakesleeana* AS 3.910 was capable of transforming betulin into BA and optimized several parameters of the fermentation process. Qazi et al. [46] also screened five kinds of fungi to select strains that were capable of transforming betulin to BA. Among *Microsporum canis, Trichophyton tonsurans, Aspergillus niger, A. niger* NIAB-280 and *Penicillium spp.*, *M. canis* and *T. tonsurans* showed strong transformation ability [46]. However, the exact BA yield was not revealed in the report. *Inonotus obliquus* was found to produce BA [47]. Some stimulating methods have been proposed to improve BA production in the liquid fermentation of *I. obliquus*. Lou et al. [48] investigated the effect of oleic acid, fungal elicitor and the combination of oleic acid and fungal elicitor on the accumulation of BA in submerged culture of *I. obliquus*. The results showed that oleic acid, fungal elicitor and their combination extremely increased the total BA levels by 78.6%, 141.6% and 404.9%, respectively, as compared to the control [48].

#### 2.3.2. Tissue Culture

Tissue culture of plants containing BA in the natural state is also a productive way to obtain BA. However, reports are scarce. The extractions of calli induced from the leaves and flowers of *Eucalyptus camaldulensis* Dehnh have been found to contain BA, along with several other functional phytochemicals [49].

### 2.4. Gene Engineering

The metabolic engineering biosynthetic pathway in microorganisms to produce terpenoids such as BA is an attractive method, and it has advantages over chemical synthesis or extraction from botanical materials. Triterpenoid is synthesized in the mevalonate pathway, in which the mevalonate transforms into farnesyl pyrophosphate (FPP) in several steps. Under the catalysis of squalene synthase, two molecules of FPP condense with reduction by NADPH to form squalene. Then, the squalene is oxidized into 2,3-oxidosqualene. The cyclization of 2,3-oxidosqualene is the first step for triterpenoid saponins synthesis [50,51,52,53,54]. Fukushima et al. [55] confirmed the BA and other triterpenoid production activity of CYP716A12 (Cyt P450 monooxygenase 716A12) from *Medicago truncatula* by expressing it in transgenic yeast. The synthesis of lupeol from 2,3-oxidosqualene was catalyzed by lupeol synthase (LUS), followed by the catalysis of CYP716A12, which was capable of carboxylation at the C-28 position to transform lupeol into betulin and BA further [55]. After that, a new CYP716A was identified and discovered to have BA synthesis capabilities [56]. Huang et al. [56] identified CYP716AL1 as the triterpene C-28 oxidase in *Catharanthus roseus*. The coexpression of CYP716AL1 from *C. roseus* and AtLUP1 from *Arabidopsis thaliana* in *Saccharomyces cerevisiae* system resulted in BA production [56]. However, linear introduction of the enzymes in the BA metabolic pathway perturbed the redox balance in cells and eventually resulted in the inhibition of cell growth [57]. Li et al. [57] constructed nine strains of yeast harboring key genes of fatty acid synthesis and BA production and modulated the carbon flux by using different promoters for those key genes. Through this measure, it was established that a higher BA production yeast with balanced carbon flux between BA synthesis and fatty acid synthesis could be constructed [57]. Considering that the cofactors NADPH and oxygen are vital for BA synthesis, Li et al. [58] managed the intracellular supply of NADPH and oxygen by expressing mutant 2,3-butanediol dehydrogenase (mBDH1) and yeast codon-optimized Vitreoscilla hemoglobin (mvhb) to modulate BA production in *S. cerevisiae*. The results showed that using acetoin with the expression of mBDH1 can increase BA production with normal cell growth, and the expression of mvhb inhibits cell growth but increases BA production [58].

## 3. Biological Activities

BA has gained a lot of concentration and considerable interest in research because of its potent physiological and pharmacological activities. It has been reported that BA has a variety of biological and pharmacological effects, including antitumor, anti-inflammatory, anti-HIV, antidiabetic and other activities such as antibacterial, antiviral, antimalarial and so on [1]. Thus, BA was considered to be a promising potential drug compound. Some of the most interesting and important biological activities are discussed below.

### 3.1. Antitumor Activity

The usual treatments for cancer are chemotherapy and surgery. However, these treatment means are always accompanied by several potential side effects. Recently, it was discovered that immunomodulatory plant products have anticancer effects, some even with no side effects. BA is considered a future promising antitumor compound and it is effective for many types of cancer (Table 1). In 1995, Pisha et al. [59] first discovered that BA is a selective inhibitor of human melanoma. Since then, the antitumor activity of BA has been extensively reported. According to reports, BA has a curative effect on leukemia, malignant head and neck cancer, colorectal carcinoma, breast tumor, lung carcinoma, hepatoma, cervical cancer, ovarian cancer and other carcinomas [5,60,61]. One study examined the cytotoxicity of BA against human promyelocytic leukemia HL-60 cells. The mean concentration of CK for inhibiting cell proliferation by 50% (IC_50_) was 5.7 μm after 72 h [62]. In addition to causing apoptosis, BA also induced HL-60 differentiation by 10% to 20%, and cotreatment of BA and 1α,25-dihydroxyvitamin D3 enhanced this differentiation [62]. The result was confirmed by a study showing the IC_50_ of BA was 2.60 μg/mL for HL-60 and 2.10 μg/mL for the WEHI-3B cell line [63]. Khan et al. [64] also found the IC_50_ (48 h) of BA toward HL-60 was 8 μm. Another study compared BA with ten kinds of standard therapeutics, and the results showed that BA was more potent than nine of the ten therapeutics [65]. The intracellular signaling pathway of BA-induced apoptosis of leukemia cells was that BA stimulated mitochondria to release cytochrome c and Smac and cause further apoptosis reactions [65]. Wu et al. [66] found that BA was cytotoxic to leukemia K-562 cells with an IC_50_ of 21.26 μg/mL at 24 h, and BA inhibited K-652 proliferation by induced apoptosis in a time- and dose-dependent manner and cell cycle arrest. Kesseler et al. [67] treated several cell lines of five kinds of cancer with BA to confirm that BA exerts cytotoxicity on cancers of different tissues. Mullayer et al. [61] developed a liposome formulation of BA to attenuate its weak hydrosolubility and delivered a BA-containing liposome into mice xenografted with human colon and lung cancer tumors. This treatment significantly reduced the tumor growth and increased the survival rate of mice with the tumor [61]. The tumor volume of colon cancer SW480 in treated mice was about 1/3 of the control [61]. The BA-containing liposome treatment reduced more than half of the tumor volume of mice with lung cancer A549 compared with the control treatment [61]. In an article studying the mechanism of BA’s antitumor effects on colon tumors, researchers treated RKO and SW480 colon cancer cell lines with BA and found that BA significantly inhibited cell growth after 5 h and induced apoptosis [68]. Researchers also concentrated on the effect of BA on the chemoresistant cell lines of colon cancer. Cell lines resistant to 5-fluorouracil (5-FU) (SUN-C5/5FU-R), IRT (SUN-C5/IRT-R) and OXT (SUN-C5/OXT-R) were isolated from the wild-type colon cancer cell line (SUN-C5/WT), and BA alone was effective against wild type, 5FU-R and OXT-R. BA combined with OXT was capable of inducing the apoptosis of the SNU-C5/OXT-R cell [69]. Aisha et al. [70] not only found that BA inhibited the growth of colon cancer cell HCT116 in a dose-dependent manner but also that the combination of 2.5 μg/mL α-mangostin and BA could significantly increase the cytotoxicity of BA. BA combined with cisplatin showed reduced cytotoxicity in subcytotoxic BA concentrations, but when the BA concentration was above 7.5 μg/mL, combined with cisplatin, 100% of colorectal cancer cells were killed.

Researchers also studied BA’s effect on the cell lines of prostate cancer. Rabi et al. [71] treated androgen-refractory human prostate cancer cells PC-3 with BA, and the cell line they used expressed high constitutive NF-κB, which partly led to the chemoresistance of androgen-refractory prostate cancer. The results revealed that the treatment of BA inhibited DNA binding and reduced the nuclear levels of NF-κB/p65 [71]. BA treatment reduced IKK activity and stimulated IκBα phosphorylation at serine 32/36 and subsequent degradation. BA’s inhibitory effect on the activation of NF-κB induced by TNFα through the IκBα pathway reduced TNFα-induced apoptosis [71]. In a study intended to depict the role of antiapoptotic protein Mcl-1 in resisting DNA damage induced by chemotherapy in prostate cancer, BA combined with ENMD-1198, an antitumor agent, significantly increased apoptotic/necrotic cell death and inhibited metastasis by decreasing Mcl-1, which led to increased DNA damage [72]. BA isolated from the flower stalks of *Prunella vulgaris* var. lilacina was reported to have a significant inhibitory effect on estrogen-mediated signaling [18]. When MCF7 breast cancer cells were treated with BA, the synthesis of estrogen-responsive gene growth regulation by estrogen in breast cancer 1 (GREB1) mRNA was significantly inhibited, ERE-dependent luciferase activity was significantly suppressed, and estrogen receptor α(Erα)-mediated signaling was suppressed by inhibiting Era mRNA synthesis to suppress the ERα protein levels.

Multiple myeloma (MM) is a type of severe cancer that has a higher disease incidence among people over 40, especially in elderly people over 60. BA is capable of inducing apoptosis in MM cells. A study treating multiple myeloma cell lines U266 and MM.1S (dexamethasone sensitive) with BA found that BA inhibited the signal transducer and activator of transcription protein 3 (STAT3) activation [73]. The induced expression of protein tyrosine phosphatase SHP-1 and the silencing of the SHP-1 gene further maintained BA-induced cell apoptosis [73]. The combination therapy of BA with thalidomide and bortezomib enhanced the apoptosis-inducing effect of chemotherapeutics [73]. One of the mechanisms through which BA selectively kills cancer cells is the alternation of mitochondrial permeability. Potze et al. [74] suggested that BA mediates mitochondrial-dependent cell apoptosis by inhibiting the activity of stearoyl-CoA-desaturase (SCD-1), which catalyzes the conversion of newly synthesized saturated fatty acids to unsaturated fatty acids, thereby influencing the saturation levels of cardiolipin (CL), which results in ultrastructural changes in mitochondrial and cytochrome c release in tumor cells. This mechanism was found in all four cells, including HeLA cells, A549 cells, MCF-7 cells and RKO cells [74].

**Table 1:** Effect of BA on different cancer cell lines.

Cancer Type	Cell Line/Animal	Dose	Reference
Leukemia	Cell line: HL-60	IC_50_ = 5.7 μm (72 h)	[62]
	Cell line: K-562	IC_50_ = 21.26 μg/mL (24 h)	[66]
	Cell line: K-562	IC_50_ = 12.5 μg/mL (48 h)	[75]
	Cell line: HL-60	IC_50_ = 2.60 μg/mL (72 h)	[63]
	Cell line: WEHI-3B	IC_50_ = 2.10 μg/mL (72 h)	
	Cell line: HL-60	IC_50_ = 8 μm (48 h)	[64]
Colorectal carcinoma	Cell line: SW1463	EC_50_ = 3.8 μg/mL (48 h)	[67]
	Cell line: SW837	EC_50_ = 11.3 μg/mL (48 h)	
	Cell line: RKO	EC_50_ = 9.5 μg/mL (48 h)	
	Cell line: CO115	EC_50_ = 12.2 μg/mL (48 h)	
	Cell line: SW480	EC_50_ = 15.1 μg/mL (48 h)	
	Cell line: T84	EC_50_ = 11.3 μg/mL (48 h)	
	Cell line: HCT81	EC_50_ = 16.4 μg/mL (48 h)	
	Cell line: LS180	EC_50_ = 11.7 μg/mL (48 h)	
	Animals: female athymic nude Foxn1 mice xenografted with human colon cancer cell line SW480.	Intravenously injected 200 μL of BA-containing liposomes containing 5 mg/mL of BA three times a week.	[61]
Lung carcinoma	Cell line: HCT116	IC_50_ = 8.9 μg/mL (48h)	[70]
	Cell line: A549	IC_50_ = 8 μm (48h)	[64]
	Cell line: H460	EC_50_ = 6.1 μg/mL (48 h)	[67]
	Cell line: A549	EC_50_ = 8.3 μg/mL (48 h)	
	Cell line: H322	EC_50_ = 12.3 μg/mL (48 h)	
	Cell line: GLC-2	EC_50_ = 8.8 μg/mL (48 h)	
	Cell line: GLC-4	EC_50_ = 10.0 μg/mL (48 h)	
	Cell line: GLC-36	EC_50_ = 9.6 μg/mL (48 h)	
	Cell line: H187	EC_50_ = 8.7 μg/mL (48 h)	
	Cell line: N417	EC_50_ = 6.2 μg/mL (48 h)	
	Cell line: MBA9812	EC_50_ = 7.6 μg/mL (48 h)	
	Animals: female athymic nude Foxn1 mice xenografted with human lung cancer cell line A549.	Intravenously injected 200 μL of BA-containing liposomes containing 5 mg/mL of BA three times a week.	[61]
Prostate cancer	Cell line: PC-3	IC_50_ = 7 μm (48 h)	[64]
	Cell line: DU145	EC_50_ = 11.6 μg/mL (48 h)	[67]
	Cell line: PC3	EC_50_ = 12.3 μg/mL (48 h)	
	Cell line: 22Rv1	EC_50_ = 10.1 μg/mL (48 h)	
	Cell line: LNCaP	EC_50_ = 11.9 μg/mL (48 h)	
Pancreatic cancer	Cell line: MiaPaca-2	IC_50_ = 7 μm (48 h)	[64]
Breast adenocarcinoma	Cell line: MCF-7	IC_50_ =20.4 μg/mL (72 h)	[63]
	Cell line: SKBR3	EC_50_ = 16.2 μg/mL (48 h)	[67]
	Cell line: MDA231	EC_50_ = 10.4 μg/mL (48 h)	
	Cell line: MDL13E	EC_50_ = 11.5 μg/mL (48 h)	
	Cell line: BT483	EC_50_ = 12.8 μg/mL (48 h)	
	Cell line: BT474	EC_50_ = 12.1 μg/mL (48 h)	
	Cell line: T47D	EC_50_ = 13.0 μg/mL (48 h)	
	Cell line: BT549	EC_50_ = 5 μg/mL (48 h)	
Brain tumor	Cell line: human glioblastoma DBTRG0.5MG	IC_50_ > 30.0 μg/mL (72 h)	[63]
Melanoma	Cell line: B16	IC_50_ > 30.0 μg/mL (72 h)	[63]
Cervical carcinoma	Cell line: HeLa	IC_50_ = 2.5 μg/mL (72 h)	[63]
	Cell line: CaSKi	EC_50_ = 9.6 μg/mL (48 h)	[67]
	Cell line: HeLa	EC_50_ = 14.3 μg/mL (48 h)	
	Cell line: SiHa	EC_50_ = 11.8 μg/mL (48 h)	

### 3.2. Anti-Inflammatory Activity

Inflammation has been regarded as the main factor of many diseases, as it can lead to cell death, organ-specific damage or some cancers. In recent years, many studies have confirmed that BA has an anti-inflammatory effect. Costa et al. [76] reported that in a mouse model of endotoxic shock, BA exhibited potent anti-inflammatory activity. Administration of BA protected all mice from a lethal dose of lipopolysaccharide (LPS), significantly inhibited tumor necrosis factor (TNF)-α release induced by LPS and increased the interleukin (IL)-10 level in serum. In vitro experiments also showed that BA treatment inhibited TNF-α and NO in LPS-activated macrophages and enhanced the production of IL-10 [76]. To evaluate the effect of BA on septic acute lung injury (ALI), Lingaraju et al. [77] set up a cecal ligation and puncture (CLP) model in mice pretreated with BA. Administration of 10 and 30 mg/kg of BA significantly improved survival against sepsis and attenuated lung injury. In addition, BA inhibited nuclear factor-kappa B (NF-κB) expression in the lung and decreased levels of cytokine, intercellular adhesion molecule-1 (ICAM-1), monocyte chemoattractant protein-1 (MCP-1) and matrix metalloproteinase-9 (MMP-9) [77]. Nader et al. [78] also assessed the effect of BA on LPS-induced ALI by evaluating neutrophil recruitment and inflammation mediators. Pretreatment of 25 mg/kg BA by oral seven days before LPS nasal instillation significantly inhibited increased lipid peroxidation, expression of tumor necrosis-α (TNF-α), transforming growth factor-b1 (TGF-b1) and inducible nitric oxide synthase (iNOS) [78]. BA pretreatment also attenuated pulmonary edema and Evans blue extravasation in lung tissue [78]. BA from *Erythrophleum ivorense* (A Chev.) was applied in chicken injected with carrageenan in the right footpad to evaluate its anti-inflammatory properties [79]. BA exhibited a significant anti-inflammatory effect by reducing carrageenan-induced edema [79]. Viji et al. [80] treated LPS-stimulated human peripheral blood mononuclear cells (hPBMCs) with BA to identify the mechanism through which BA exhibits anti-inflammatory effects and employed mice of LPS-induced endotoxin shock as an in vivo model. In hPBMCs, BA suppressed cyclooxygenase-2 (COX-2) expression and prostaglandin E_2_ (PEG_2_) production by inhibiting extracellular regulated kinase (ERK) and Akt phosphorylation and thereby modulated the NF-κB signaling pathway [80]. Interestingly, BA significantly decreased the mortality of mice against endotoxin shock and inhibited the production of PEG_2_ in two of the most susceptible organs, lungs and livers [80]. Moreover, BA reduced reactive oxygen species (ROS) formation and the release of lactate dehydrogenase [80]. Another experiment that evaluated the in vitro inhibitory effect of BA on bovine prostaglandin synthase showed that 200 μg/mL BA inhibited the enzyme by 52% [81]. Recio et al. [82] assessed the anti-inflammatory effect using a model of 12-O-tetradecanoylphorbol-13-acetate (TPA)-induced ear edema, and BA at a dose of 0.5 mg/ear significantly reduced edema by 86.2%. BA administered orally at a dose of 10 mg/kg reduced inflammation by 45.6% [82]. In a similar study, 0.5 mg/ear of BA resulted in a decrease in neutrophil infiltration by 29% [83]. In a mice model with paw edema induced by carrageenin and serotonin, oral administration of BA at 50 and 100 mg/kg significantly reduced paw edema [84]. Krogh et al. [85] extracted BA from *Ipomoea pescaprae* (L.) R. Br. and assessed its antinociceptive and anti-inflammatory properties. It was found that pretreatment of BA at a dose of 10 mg/kg attenuated inflammatory pain by 50% in mice whose paws were injected with formalin [85]. BA from *Prunello vulgaris* modestly inhibited the nitric oxide production in cultured murine macrophage RAW 264.7 cells [86]. Huguet et al. [87] tested 11 natural compounds on their anti-inflammatory effects; among them, BA inhibited mouse ear edema induced by mezerein-, 12-deoxyphorbol-13-tetradecanoate (DPT) and 12-deoxyphorbol-13-phenylacetate (DPP) by 48%, 51% and 61%, respectively. Additionally, 1 h pretreatment of BA of bradykinin-induced mouse paw edema caused significant inhibition by 54% [87].

### 3.3. Anti-HIV Activity

BA isolated from the leaves of *Syzigium claviflorum* was reported to have an inhibitory effect against HIV replication in H9 lymphocyte cells [88]. This study presented that BA inhibited HIV replication in H9 cells with an EC_50_ of 1.4 μm but exhibited inhibition against C9 cell growth with an IC_50_ of 13 μm [88]. Reverse transcriptase (RT) was vital for the replication of HIV; thus, inhibition against RT is thought to be a promising prophylactic method against acquired immunodeficiency syndrome (AIDS). Akihisa et al. [89] screened 55 triterpenoids and found that 20 triterpenoids showed inhibitory activities on purified HIV-1 with IC_50_ values less than 5.0 μm, including BA. In addition, BA was shown to be a potent HIV-1 RT inhibitor [89]. Many studies have focused on the anti-HIV effect of BA derivatives. These activities include blocking the entry of HIV into cells [90] and inhibiting HIV protease [91], which will be elaborated on later.

### 3.4. Antidiabetic Activity

Diabetes is a common disease in today’s society and leads to a great number of deaths and disabilities. Patients mainly suffer from type 2 diabetes mellitus (T2DM). BA is considered a novel antidiabetic agent. Kim et al. [92] demonstrated that BA attenuates hyperglycemia by inhibiting hepatic glucose production through modulation of the CAMKK-AMPK-CREB pathway. In vitro, BA significantly reduced hepatic glucose production, activated p-adenosine 5′-monophosphate-activated protein kinase (AMPK) and inhibited the expression of phosphorylated cAMP response element-binding protein (CREB). In vivo, BA decreased the plasma glucose, triglyceride, and insulin resistance index in high-fat diet-fed ICR mice [92]. In addition, BA has protective effects on some diabetes complications. Yoon et al. [93] treated diabetic apolipoprotein-E gene knockout mice with BA, and the results indicated that BA has positive effects on early atherosclerosis. BA treatment for 12 weeks resulted in lower systolic blood pressure, blood urea nitrogen, triglyceride and total cholesterol levels [93]. BA-treated mice also had better blood glucose, insulin, glucose-tolerant results and homeostasis model assessment of insulin resistance index. Atherosclerotic lesions such as roughened endothelial layers were attenuated [93]. At the molecular level, BA downregulated the reduction of endothelial nitric oxide synthase (eNOS) expression, which resulted in the following inhibition of intracellular adhesion molecule 1 (ICAM-1) and endothelin 1 (ET-1) expression [93]. Diabetic nephropathy induced by intraperitoneally injected streptozotocin was also studied. Xie et al. [94] demonstrated the protective effect of BA on rats with diabetic nephropathy. Intragastric administration of BA significantly decreased the inflammatory cytokines such as IL-6, IL-1β and TNF-α in blood and kidney tissue. The histopathological condition of the kidney was ameliorated, and the activities of SOD and CAT were boosted [94]. The phosphorylation of AMPK, NF-κB and IκBα and the expression of NF-E2-related factor 2 (Nrf2) and heme oxygenase (HO)-1 in renal tissue were also reduced by BA treatment [94]. Another similar study using male mice with streptozotocin–nicotinamide (STZ-NA)-induced diabetic nephropathy also revealed the preventive effects of BA [95]. Compared to diabetic mice, BA-treated diabetic mice have increased blood albumin levels and decreased blood urea nitrogen, plasma creatine and renal histopathology levels [95]. However, Ahangarpour et al. [96] studied the effects of BA on the male reproductive system of STZ-NA-induced diabetic mice and found that BA had negative effects on the male reproductive system. BA treatment on diabetic mice even resulted in greater plasma testosterone level reduction, a higher seminiferous tubule vacuole number and a smaller diameter of seminiferous tubules than the diabetes group [96]. Wang et al. [97] also reported that BA ameliorates diabetic renal inflammation and fibrosis. BA suppressed fibronectin expression in high-glucose-induced mesangial cells and kidneys of diabetic rats by inhibiting NF-κB activity and the degradation of IκBα. BA also decreased the DNA binding activity and transcriptional activity of NF-κB in high-glucose-induced glomerular mesangial cells and increased the interaction between IκBα and β-arrestin2 in mesangial cells [97].

### 3.5. Antimalarial Activities

Malaria is one of the most important tropical diseases that has a high morbidity and mortality rate and mainly affects the population and economic development of developing countries. In early 1999, BA was evaluated on its antiplasmodial effects [98]. Steele et al. [98] isolated BA from the root bark of Uapaca nitida Mu¨ll-Arg. (Euphorbiaceæ) and tested its in vitro and in vivo antimalarial effects. The IC_50_ values of BA against chloroquine-resistant (Kl) and chloroquine-sensitive (T9-96) *Plasmodium falciparum* were 19.6 and 25.9 μg/mL. De Sa et al. [99] studied the antimalarial activity of BA and its derivatives and found that they showed antiplasmodial activity against chloroquine-resistant *Plasmodium falciparum* parasites in vitro. The IC_50_ value of BA was 9.89, and treatment of mice infected with *Plasmodium berghei* with BA showed a dose-dependent decrease in parasitemia. This indicates that BA is a candidate for the development of new antimalarial drugs. Olanlokun et al. [100] purified BA from *Alstonia boonei* and found that BA demonstrated antiplasmodial activities using chloroquine susceptible (NK 65) and resistant (ANKA) strains of *Plasmodium berghei*. In a previous study, *Plasmodium* infection led to liver mitochondrial pathology, accompanied by nonselective apoptotic effects of antimalarial drugs in liver mitochondria [101]. BA opened mitochondrial permeability transition pore (mPT), increased mitochondrial F_1_F_0_ ATPase activity and decreased lipid peroxidation and DNA fragmentation. Additionally, the authors found that BA mechanistically prevented and disrupted the secondary structure of mATPase (mouse) with RMSD > 2 (RMSD = 2.34 ˚A) by molecular dynamics simulation studies against falcipain-2, dihydrofolate reductase, dihydropteroate synthase and mitochondrial F_1_F_0_ ATPase (F-2- BA, DHFR- BA, DHPS- BA and mATPase- BA, respectively) [100]. Furthermore, some BA derivatives have been studied for antimalaria effects [102,103]. A series of BA /betulin-based dimer and hybrid compounds were analyzed in vitro against malaria parasites and human cytomegalovirus (HCMV), and Karagoz et al. found that the BA /betulin and artesunic acid hybrids 11 and 12 showed the most potent activities against *P. falciparum* and HCMV [102].

### 3.6. Other Activities

BA has been reported to have several other bioactivities beneficial to multiple organs. Several papers have indicated that BA attenuates ethanol-induced liver damage. Szuster-Ciesielska et al. [104] tested the effect of betulin and BA on ethanol-induced hepatic stellate cell activation. Preincubation with 1 μM BA significantly suppressed ethanol-induced ROS production and migration in hepatic stellate cells (HSCs) and also inhibited TNF-α production [104]. Significant inhibition of TGF-b production was observed after 24 h of preincubation with 1 or 5 μM BA [104]. BA decreased the ethanol-induced metalloproteinase-2 (MMP-2) expression in a concentration-dependent manner and downregulated tissue inhibitors of metalloproteinases (TIMP-1 and TIMP-2) modestly [104]. BA suppressed c-Jun N-terminal kinase (JNK) signal transduction and inhibited the nuclear factor-κB (NFκB) pathway by inhibiting the phosphorylation of NFκB and IκB [104]. The phosphorylation of Smad 3 was inhibited by BA and thereby suppressed tumor growth factor-β1 (TGF-β1) signaling [104]. Jain et al. [105] assessed the hepatoprotective effect of BA from *Tecomella undulata*. Pretreatment of BA prevented the depletion of hepatic antioxidants superoxide dismutase (SOD) and catalase (CAT), reduced glutathione (GSH) and ascorbic acid (AA) and decreased the CCl_4_-induced LPO level [105]. BA also attenuated the elevation of aspartate aminotransferase (AST) and alanine aminotransferase (ALT) plasma level, as well as CCl_4_-induced cellular changes, including extensive vacuolation, centrilobular necrosis and nuclear condensation [105]. In another mice model, of which liver damage was induced by alcohol, pretreatment of BA also exerted hepatoprotective effects by improving the redox system in the liver [106]. Wan et al. [107] induced liver fibrosis in Wistar rats by intraperitoneal injection of 200 mg/kg thioacetamide (TAA) and used it as a model to evaluate the effects of BA. Intragastric administration of 20 or 50 mg/kg BA every day increased serum ALT and AST and significantly attenuated hepatic hydroxyproline [107]. Moreover, BA reduced the expression of α-smooth muscle actin (α-SMA) and collagen-I [107]. BA’s hepatoprotective effects are not only underlain by preventing the liver damage induced by chemicals but also by antiviral activities. BA extracted from *Avicennia marina* was tested for its hepatitis C virus (HCV) suppression effects [37]. BA inhibited HCV replication in Ava 5 replicon cells and in a cell-culture-derived infectious HCV particle system by reducing the phosphorylation of NF-κB and ERK1/2 of the MAPK pathway, which led to the suppression of COX-2 expression [37].

BA also exhibits renal-protective effects. Renal fibrosis is an end-stage renal disease symptom that develops from chronic kidney disease (CKD). In this situation, abnormal accumulation of the extracellular matrix leads to loss of kidney tissue and function [108]. In induced chronic kidney disease rats, BA treatment significantly reversed the histological changes and abnormal regulation of the metabolic pathway induced by CKD [109]. The upregulated profibrotic protein levels in kidney tissue of CKD rats such as transforming growth factor β (TGF-β), connective tissue growth factor (CTGF), hydroxyproline, collagen type I and fibronectin were inhibited by BA treatment [109]. Dilatation of tubules and glomerular degeneration and vacuolation with deposition of collagen fibers were attenuated by BA [109]. Xia et al. [110] reported that BA had cardioprotective effects against myocardial ischemia-reperfusion injury (MIRI). In an open-chest anesthetized rat model with MIRI induced by left anterior descending occlusion followed by reperfusion, BA treatment reduced serum creatinine kinase (CK) and lactate dehydrogenase (LDH) levels, attenuated the apoptosis of cardiomyocytes demonstrated by TUNEL assay results and decreased the Bax/Bcl-2 ratio [110]. Ischemia-reperfusion injury also occurs in cerebral tissues and causes neuronal injury. BA protected against this ischemia-reperfusion injury in a mice model by enhancing blood flow and reducing oxidative stress and nitrosative stress [111]. Moreover, BA has shown therapeutic potential in treating hypothyroidism, an endocrine disorder prevalent in male and female adults. Oral administration of BA in propylthiouracil (PTU)-induced hypothyroidism in female rats decreased the thyroid-stimulating hormone level increased by PTU and attenuated histopathological changes in thyroid follicles [112].

Furthermore, BA extracted from *Rosmarinus officinalis* L. has shown antidepressant-like effects [30]. BA administered by the oral route significantly decreased the immobility time in the tail suspension test and did not present any significant differences in the locomotion of the open-field test, suggesting that BA may have a promising anti-immobility effect [30]. Jine et al. [113] suggested that BA is a potential biological response modifier and may have an immune-enhancing effect. Oral administration of BA increased the total number of lymphocytes in immune organs and modified the ratio of T-cell subsets in mice. In sheep, red blood cell immunized mice, orally administered 5 mg/kg BA five times in 24 h, increased the number of plaque-forming cells but inhibited the production of anti-SRBC antibodies on the fourth day after immunization [113]. Kim et al. [114] reported that BA exerted antiobesity effects by influencing the absorption of lipids from the small intestine by inhibiting pancreatic lipase and accelerating lipolysis in adipose tissues. In addition to these bioactivities, BA has been reported to be useful in aquaculture. The bioactive component of *Souroubea sympetala*, betulinic acid, significantly reduced the cortisol response to net confinement of rainbow trout *Oncorhynchus mykiss* and attenuated the cortisol response to adrenocorticotropic hormone of the head kidney tissue in vitro [115].

## 4. Conclusions

BA has a variety of biological activities and low toxicity, and it is a very valuable natural product. Recent studies have shown that BA has many biological activities, such as antitumor, anti-HIV, anti-inflammatory, antibacterial, antimalaria effects and so on. Particularly, betulinic acid exhibits potent activities in antitumor aspects; hence, it has attracted widespread attention and seems a promising experimental antitumor drug. In recent times, the research on the preparation of BA has gradually increased with the continuous development of synthetic biology and basic genetic engineering. More and more methods have also been discovered to prepare BA to solve supply problems.

## Data Availability

Data sharing not applicable. No new data were created or analyzed in this study. Data sharing is not applicable to this article.

## Sample Availability

Samples of the compounds are not available from the authors.
